# The Impact of Lifestyle on Prostate Cancer: A Road to the Discovery of New Biomarkers

**DOI:** 10.3390/jcm11102925

**Published:** 2022-05-22

**Authors:** Catarina Leitão, Bárbara Matos, Fátima Roque, Maria Teresa Herdeiro, Margarida Fardilha

**Affiliations:** 1 Department of Medical Sciences, Institute of Biomedicine (iBiMED), University of Aveiro, Campus Universitário de Santiago, 3810-193 Aveiro, Portugal; catarinaileitao@ua.pt (C.L.); teresaherdeiro@ua.pt (M.T.H.); 2 Cancer Biology and Epigenetics Group, IPO Porto Research Center (CI-IPOP), Portuguese Institute of Oncology of Porto (IPO Porto), 4200-072 Porto, Portugal; barbaracostamatos@ua.pt; 3 Laboratory of Signal Transduction, Department of Medical Sciences, Institute of Biomedicine—iBiMED, University of Aveiro, 3810-193 Aveiro, Portugal; 4 Research Unit for Inland Development, Polytechnic of Guarda (UDI-IPG), Avenida Doutor Francisco Sá Carneiro, 6300-559 Guarda, Portugal; froque@ipg.pt; 5 Health Sciences Research Centre, University of Beira Interior (CICS-UBI), Av. Infante D. Henrique, 6200-506 Covilhã, Portugal

**Keywords:** prostate cancer, lifestyle patterns, inflammation, biomarkers

## Abstract

Prostate cancer (PCa) is one of the most common cancers among men, and its incidence has been rising through the years. Several risk factors have been associated with this disease and unhealthy lifestyles and inflammation were appointed as major contributors for PCa development, progression, and severity. Despite the advantages associated with the currently used diagnostic tools [prostate-specific antigen(PSA) serum levels and digital rectal examination (DRE)], the development of effective approaches for PCa diagnosis is still necessary. Finding lifestyle-associated proteins that may predict the development of PCa seems to be a promising strategy to improve PCa diagnosis. In this context, several biomarkers have been identified, including circulating biomarkers (CRP, insulin, C-peptide, TNFα-R2, adiponectin, IL-6, total PSA, free PSA, and p2PSA), urine biomarkers (PCA3, guanidine, phenylacetylglycine, and glycine), proteins expressed in exosomes (afamin, vitamin D-binding protein, and filamin A), and miRNAs expressed in prostate tissue (miRNA-21, miRNA-101, and miRNA-182). In conclusion, exploring the impact of lifestyle and inflammation on PCa development and progression may open doors to the identification of new biomarkers. The discovery of new PCa diagnostic biomarkers should contribute to reduce overdiagnosis and overtreatment.

## 1. Introduction

Prostate cancer (PCa) is the 2nd most common (14.1%) and 5th deadliest (6.8%) cancer among men all over the world [1]. In 2020, the number of PCa cases was above 1.4 million, with a higher incidence in Europe, Asia, and Northern America. Moreover, the number of deaths exceeds 350,000, the highest mortality rate in Asia, Europe and Latin America, and the Caribbean [1]. In Portugal, PCa represents 20% of the estimated new cancer cases and 10.5% of the cancer-associated mortality [1]. Although PCa incidence has increased in recent years, a good prognosis is presented for many PCa patients [2].

The exact aetiology of PCa is difficult to determine, but several risk factors have been described. The major risk factor for PCa is the patients’ age, as most of the cases are diagnosed in men over the age of 50 [3]. The ageing-associated chronic inflammation seems to contribute to the increased PCa risk and underlies at least 20% of all human cancers [4]. Other known risk factors include family history of the disease [3], genetic alterations [5], high serum levels of insulin-like growth factor 1 (IGF-1) [5], and black ethnicity [6]. In contrast, several studies associated a healthy lifestyle with a decline in the incidence, development, and severity of PCa [7,8,9]. A healthy lifestyle, represented by the Mediterranean diet (MedDiet) and regular physical activity [9,10,11], has been associated with improved survival and decreased PCa progression [12,13]. The use of some medicines (5-α reductase (5-AR) inhibitors [14,15], nonsteroidal anti-inflammatory drugs (NSAIDs) [16], and statins [17]) was also associated with improved survival [12,13]. In contrast, dairy products with higher calcium content, alcohol consumption, smoking, sexually transmitted diseases (STDs) [13,18,19,20], higher waist circumference associated with abdominal obesity, weight gain, higher body mass index (BMI) [21,22,23], hypercholesterolaemia [24,25], hypertriglyceridaemia [26], and prostatitis [18,27] were positively correlated with the risk of advanced PCa.

Herein, we explore the impact of lifestyle and inflammation on PCa, with the ultimate goal of identifying putative novel biomarkers that may enable an earlier and more effective PCa diagnosis. Hopefully, the identified biomarkers may also be beneficial in the prevention of the disease.

## 2. Prostate Cancer (PCa) Diagnosis

### 2.1. Traditional Diagnosis Methods

For the last several years, PCa has evolved from a rare disease to one of the most common cancers all over the world [28], which is mainly explained by the recent improvement in diagnostic methods. The identification of biomarkers such as prostate-specific antigen (PSA) has allowed a better understanding of how PCa incidence is distributed around the world. PSA is a serine protease of the Kallikrein family produced by the prostate and it is a component of the seminal fluid crucial for semen ejaculation [29]. Since the implementation of PSA screening for PCa diagnosis, most countries registered a significant increase in PCa incidence [13]. However, using PSA screening as an early detection method for PCa remains an uncertain and controversial strategy. There is a large percentage of screen-detected cancers that do not present any symptoms, which often leads patients to receive unnecessary treatment [30]. This is extremely relevant because PCa treatment presents several side-effects: sexual impotence, urinary incontinence, and bowel syndrome. Thus, to contemplate PSA screening, patients should consider the main risk factors associated, such as the advanced age, family history of PCa, and ethnicity [30]. The recommendations for PSA screening are: (i) initial PSA screening with or without digital rectal examination (DRE); (ii) for PSA levels below 2.5 ng/mL, screening intervals can be extended to every 2 years; (iii) annual screening for men whose PSA level is equal or above 2.5 ng/mL; (iv) for PSA levels between 2.5 ng/mL and 4.0 ng/mL, healthcare providers should consider an individual risk assessment that incorporates high-grade cancer, and may lead to a biopsy recommendation; (v) for PSA levels equal or above 4.0 ng/mL or above, referral for further evaluation or biopsy is suggested, which remains a reasonable approach for men at average risk for PCa [30].

DRE is an examination of the lower rectum, pelvis, and lower abdomen that is also commonly used for PCa diagnosis, either alone or to complement PSA screening. Similar to PSA serum levels, DRE can also result in a high number of false positive results, which can lead to unnecessary invasive diagnostic tests, as well as overdiagnosis and overtreatment [31,32,33,34]. Therefore, a combination of PSA screening and standardised DRE procedures, along with patients’ history, may improve accuracy and minimise overdiagnosis of lethargic PCa.

### 2.2. Recent Diagnosis Methods

In the last decade, many studies have contributed to improve the diagnosis of PCa. One of the methodologies that recently emerged to diagnose PCa was the magnetic resonance imaging/transrectal ultrasound (MRI/TRUS) fusion-guided biopsy. MRI/TRUS can identify PCa patients with previous negative biopsies and clinically insignificant tumours, preventing overtreatment. Mainly limited by the economic resources that would be required, MRI/TRUS has been considered a significant diagnostic tool in PCa [35].

Another technique that uses MRI is the multiparametric prostate MRI (mp-MRI). This is a promising tool that may help prevent overdiagnosis of insignificant PCa. Incorporating this technique into the clinical pathway of PCa detection is still an ongoing process, as the development of a standardised reporting system is the biggest challenge due to biased classification. When abnormal values occur, mp-MRI is linked to larger tumour volume and higher tumour grade [36]. According to the PCa guidelines of the European Association of Urology (EAU), mp-MRI could be used in two different procedures: (i) to improve PCa detection by combining a targeted biopsy with systematic biopsies for positive mp-MRI results and conducting systematic biopsies alone for negative mp-MRI results, and (ii) to perform a pre-biopsy triage test, in which a targeted biopsy alone would be executed only for positive mp-MRI results [37]. Therefore, integrating this modality into diagnostic procedures may help reduce both overdiagnosis and underdiagnosis [38]. The use of microRNAs (miRNAs) [39] and Prostate-Specific Membrane Antigen (PSMA) [40] are gaining relevance, not only for PCa diagnosis but also for therapeutic purposes. The miRNAs regulate gene expression by partially binding to their target mRNAs, resulting in the inhibition of mRNA translation or degradation of the target mRNA. They can be measured in both prostate tissue or body fluids (circulating miRNAs), including serum, plasma, saliva, urine, and seminal fluid, preventing the necessity of a biopsy [39]. Furthermore, because miRNAs regulate gene expression, they are extremely important in tumour development follow-ups. Thus, they can be used to determine the most appropriate treatment regimen, predict the response to a specific treatment, or enhance the tumour’s sensitivity to other therapies. A clinical trial is currently in progress (ClinicalTrials.gov Identifier: NCT04188275) to evaluate the efficacy of circulating miRNAs in assessing treatment efficiency in PCa. Nevertheless, the usefulness of miRNAs for PCa diagnosis still requires clinical validation [41,42].

PSMA is a transmembrane protein located on the apical side of the prostatic epithelium in the cytoplasm of a benign cell. When a malignant transformation occurs, PSMA is translocated to the luminal surface of the prostatic ducts, where it forms a large extracellular domain to bind ligands [43]. The expression of this molecule has been demonstrated in many malignancies, including renal cell carcinoma, urothelial carcinoma, breast cancer, and colonic cancer [44,45]. Although it is common in salivary glands, duodenal mucosa, proximal renal tubular cells, and neuroendocrine cells in the colonic crypts, its expression is much lower compared to PCa lesions and it generally rises with tumour dedifferentiation [46]. Studies have shown that there is an increased expression of PSMA in most cases of PCa, especially in metastatic cancers [47,48]. However, the uptake of PSMA-targeting radiotracers into these normal tissues has been associated with toxicity, and in the absence of metastases, its uptake is nonexistent in the lymph nodes and bone [49]. The use of PSMA-targeted imaging by fluorine-18 and ^68^Gallium-labelled compounds has already allowed PCa lesions to be detected at higher rates than conventional imaging technologies for low-volume PCa lesions [46].

Alternatively, as a surface-sensitive technique, surface-enhanced Raman scattering (SERS) spectroscopy can improve the Raman scattering intensity produced by the molecules upon adsorption on rough metal surfaces or by nanostructures such as plasmonic-magnetic silica nanotubes [50]. When patients with PCa were compared to those with high serum PSA levels, studies showed that combining PSA levels and SERS spectra acquired from serum samples increased the accuracy of PCa detection [51]. Moreover, with a pattern recognition technology, it can also help to understand the serum levels of tumour markers, enhancing diagnostic accuracy [52].

## 3. Impact of Lifestyle on PCa Development

As there is high variability in the incidence of PCa between races, genetic variations and polymorphisms were believed to be the only risk factors associated with this disease. However, when Japanese men emigrated to Western countries, it was revealed that they had higher incidences of PCa compared with those that remained in Japan [53,54]. This suggests that other factors contribute to PCa development. In addition to innate factors, environmental factors, such as differences in eating habits, increased BMI, and obesity, have also been strongly associated with PCa [55].

### 3.1. Eating Habits

The Western diet is characterised by high intakes of red meat, processed foods, ‘‘fast-foods’’, high-fat dairy products, snacks, and sugary soft drinks, and low intakes of fruits, vegetables, vitamins, and minerals [56]. This type of diet is often associated with a high incidence of PCa, as well as an increased propensity to severe stages of the disease [57,58]. Furthermore, red and processed meat have been positively linked with a higher incidence and mortality from many types of cancer, including PCa [59,60].

Carbohydrates are macronutrients that provide glucose to the body, which is converted into energy, that is used to support body functions [61]. Although they are necessary for a healthy diet, the source of carbohydrates can vary. The healthiest sources of carbohydrates are unprocessed or minimally processed grains, vegetables, fruits, and beans [61]. These promote good health by delivering vitamins, minerals, and fibres. On the other hand, unhealthier sources of carbohydrates include white bread, pastries, soft drinks, and highly processed or refined foods [61]. These contain easily digested carbohydrates that contribute to weight gain, interfere with weight loss, and promote diabetes and heart diseases. Different studies [62,63] have evaluated the hypothesis that reducing these macronutrients may slow PCa development, by decreasing serum insulin or altering insulin-like growth factor (IGF). IGF is responsible for mitogenic and antiapoptotic effects on prostate epithelial cells. In castrated mice that mimic advanced stages of PCa, a low-carbohydrate diet reduced prostate tumour growth, compared to a Western diet [64,65]. Clinical studies with PCa patients also confirmed these findings, demonstrating that a high intake of refined carbohydrates was associated with an increased PCa risk [57,65] and that a low-carbohydrate, high-protein diet was associated with a lower PCa incidence [66].

This is not observed when a Mediterranean diet (MedDiet) is followed. Unlike the Western diet pattern, the MedDiet is characterised by a decreased consumption of saturated animal fat (no more than 8% of total caloric intake) and red meat, and a higher intake of plant-based foods (fruits, vegetables, bread, other cereals, potatoes, beans, nuts, and seeds). Olive oil is the main source of fat and dairy products such as yoghurt and light cheese; fish and poultry may be consumed in low-to-moderate amounts and egg consumption is restricted to four units per week [67]. This type of diet has been frequently linked to a significantly lower risk of overall malignancies, especially colorectal cancer, pharyngeal and oesophageal cancer, and PCa. This protective effect is accomplished because the whole food pattern can suppress spontaneous mutations, regulate cell proliferation mechanisms, methylation of DNA, and apoptosis [67]. Furthermore, a meta-analysis evaluated the role of tomatoes and lycopene (a component of tomatoes) in PCa [68]. The authors found that tomato products may reduce the risk of developing PCa because lycopene exerts antioxidant properties in downregulating mechanisms involved in the inflammatory response [68]. Moreover, olive oil exerts its beneficial effects due to its content of monounsaturated fatty acids, mainly oleic acid, and phenolic antioxidants such as phenols and flavonoids. Vegetables and fruits also have a high content of flavonoids, which are known for their antioxidant activity, and anti-mutagenic and anti-proliferative properties [67]. Omega-3 polyunsaturated fatty acids (PUFAs), which are present in fish and nuts, have also demonstrated a protective effect against PCa, by delaying tumour development and progression [69].

### 3.2. Physical Activity

Physical activity (PA) has been identified as beneficial in reducing the risk of several diseases, especially cardiovascular, musculoskeletal, pulmonary, and neurological [70]. However, regarding PCa, the topic is still under debate. A review of 83 studies conducted between 1996 and 2016 reported contradictory results. While 7 of them showed an increased incidence of PCa with PA, 31 found no association, and 45 studies demonstrated a trend or significant risk reduction of up to 30% [71]. Despite these conflicting results, regular PA appears to play a beneficial role in PCa, by preventing disease development and progression, and improving treatment outcomes. The contradictory results might be explained by the existence of different types of exercise training and intensity variation across studies.

In a prospective cohort study [72], a strong inverse association was observed between walking pace after diagnosis and the risk of PCa progression. Indeed, men who walked briskly for 3 or more hours/week had the lowest risk of progression. There was also a tendency for an inverse connection with intense activity, but the clinical sample was not significant. Brisk walking may affect PCa progression by reducing insulin resistance, decreasing bioavailable IGF-1, and increasing adiponectin levels. A role of circulating levels of insulin, bioavailable IGF-1, and adiponectin in PCa cell proliferation and apoptosis in vitro [73,74,75] and in vivo [73] has been suggested and associated with a higher risk of advanced PCa [76,77]. In another prospective cohort study [78], it was reported that men diagnosed with stage II-IV PCa, who survived at least two years after PCa diagnosis, and who became more physically active postdiagnosis or performed more recreational PA before and after diagnosis, survived longer. Furthermore, PA was also advised for patients to alleviate treatment-related side effects and improve quality of life, especially for patients receiving ADT [79,80]. Men with localised PCa who performed exercise training, such as walking/bicycling over 20 min/day, household work over 1 h/day, or exercising over 1 h/week, were associated with lower overall mortality rates. In addition, a longer time spent walking or bicycling was also reported to decrease PCa-specific mortality rates [81].

### 3.3. Alcohol Consumption

Alcohol consumption is considered one of the most addictive behaviours and has been reported as one of the most important risk factors for human cancers [82,83,84,85]. In colorectal cancer, heavy alcohol consumption was positively associated with increased risk, compared with light-to-moderate drinking in a meta-analysis of 16 cohort studies [86]. However, when it comes to PCa, the effect of alcohol consumption shows conflicting results.

A meta-analysis, from 2000, reported no association between alcohol consumption and PCa development [87], but in subsequent studies, increased PCa risk was related to higher levels of alcohol consumption [88,89,90]. In recent years, diverse meta-analyses have found that there is a strong relationship between the amount of alcohol consumed and PCa risk and mortality [83,91]. Moreover, heavier drinking and heavier alcohol exposure earlier in life were shown to be associated with an increased risk of high-grade disease, but had no association with low-grade PCa risk [92,93]. Red wine has been associated with a reduction of PCa risk, especially in more aggressive forms of the disease [94,95]. Despite inconsistent results, there is a tendency to associate alcohol consumption and earlier life exposures with higher PCa risk.

### 3.4. Smoking

Aside from alcohol, smoking is one of the most common additive behaviours and it is a well-established cause of lung cancer. Smoking is also a risk factor for other cancers, including bladder, kidney, gastrointestinal tract, and cervical cancers [96,97,98].

Regarding PCa, while recent reviews have reported no association between tobacco smoking and PCa incidence [99,100], the same did not happen with PCa mortality. Indeed, tobacco smoking could potentially play a role in PCa progression [100]. However, as with alcohol consumption, the results are contradictory. In PCa studies, smoking assessment is difficult, as they vary from smoking status at interview [101,102,103] to age at smoking onset [102,104], total consumption time [103,104], number of cigarettes [101,102], years since smoking cessation [102,105], and smoking index (pack/years) [103,104,105]. The last one is considered the gold standard for smoking assessment. Yet, it still provides inconsistent results, as the frequency, duration, and intensity of smoking may vary throughout life [106]. Cigarette smoking has been correlated with aggressive and advanced PCa in non-African American men, and there is increasing evidence that smokers have worse treatment responses [107]. Several cohort studies indicated that smokers are at lower risk for a PCa diagnosis [108,109,110,111], while other studies showed the opposite results [112,113,114,115]. Smoking was also associated with more advanced tumour stages and more aggressive baseline disease characteristics [102,110,116,117,118]. Still, further studies are needed to understand the specific role of smoking on PCa.

### 3.5. Use of Medication

Multiple medications have been studied for their role in PCa primary prevention, such as proton pump inhibitors (PPIs) [119], statins and NSAIDs [120], and secondary prevention, such as 5-AR inhibitors [121] and alpha-blockers [122].

#### 3.5.1. Primary Prevention

PPIs are known to inhibit acid secretion and were originally developed to inhibit the extrusion of protons through H^+^/K^+^ ATPases in stomach cells [123,124]. They have also been associated with a reduction in Vacuolar-type H^+^ ATPase (V-ATPase) activity [119]. This reduced activity has been shown to have an anticarcinogenic effect in breast cancer [125], PCa [126], and melanoma [127]. However, recent studies have not demonstrated a chemopreventive effect of PPIs in PCa [119,128].

Statins are mainly used to correct lipid profiles and reduce cardiovascular morbidity and mortality [129]. Furthermore, statins may also have a chemopreventive role in cancer, by limiting cancer cells proliferation, through decreasing cholesterol availability [120]. Recent studies have confirmed the chemoprotective effect of statins, and an association with decreased PCa progression and mortality has also been described [120,130,131].

NSAIDs are a family of drugs used for their analgesic and antipyretic properties, ultimately inhibiting inflammation. The well-recognised target of these drugs is the cyclooxygenase enzyme activity of both COX-1 and COX-2 receptors [132,133,134]. As chronic inflammation has been described as one of the potential risk factors for PCa, it is important to avoid its development. Therefore, aspirin and NSAIDs have been suspected to have a preventive effect against PCa. However, the evidence is limited and still controversial. A population-based cohort study from 2017 [134] suggests that there is a decreased risk of PCa in patients treated with aspirin and NSAIDs, mainly when taken over a longer period. An EPICAP (EPIdemiology of Prostate CAncer) study from 2017 [132] also found a negative association between NSAIDs use and PCa incidence, reporting a 23% reduction in PCa risk. This effect was also assured at higher doses and exposure. A meta-analysis from 2018 [135] confirmed these results and demonstrated that NSAIDs and aspirin have a protective effect on PCa development, especially with longer exposures.

#### 3.5.2. Secondary Prevention

5-AR inhibitors are usually used to treat benign prostatic hyperplasia (BPH) and they include finasteride and dutasteride [121]. The use of these type of medicines prevents the intraprostatic conversion of testosterone into dihydrotestosterone, which is a strong androgen. This allows the reduction of prostate volume and improvement of urinary outflow obstruction. Moreover, 5-AR inhibitors can reduce PSA levels by approximately 50% [136]. However, if PCa has not yet been diagnosed, taking this medicine may result in a delayed diagnosis, which may worsen the PCa outcome. Apart from this single recognised limitation, 5-AR inhibitors are suggested to be beneficial in preventing PCa development and progression [137,138,139].

Alpha-blockers are clinically used for hypertension and BPH, and they target α-adrenoreceptors. The most common α1-adrenoreceptor is present in the smooth muscle cells of the prostate gland and bladder neck. This type of receptor is responsible for decreasing smooth muscle tone and relieving bladder obstruction secondary to periurethral prostatic enlargement [122,140,141]. The α1-blocker quinazoline has been associated with anticancer activities, by preventing tumourigenesis, as well as mitigating progression to metastatic disease, by targeting anoikis and angiogenesis [122]. Moreover, prazosin, a quinazoline-based drug, in high doses can significantly reduce the risk of PCa recurrence and delay the time to biochemical relapse in PCa patients following radiotherapy [142].

### 3.6. Sexual Behaviour and STDs

There is evidence that STDs and sexual behaviour, including the number of sexual partners, age at first sexual intercourse, and frequency of sex intercourse, influence the risk of developing PCa [13,18,19,20,143].

In a 2014 study, while the age at which men had their first intercourse did not display an increased risk of PCa, the number of sexual partners (female and male) led to different results [20]. Men who were with more than 20 female sexual partners had decreased PCa risk, whereas a higher number of male sexual partners increase it [20]. This suggests that PCa risk is higher for bisexual and homosexual men. Similar conclusions have been reported regarding STDs [144,145]. However, some studies report a beneficial role of more frequent ejaculation in adulthood, particularly for low-risk PCa [13,146,147].

A meta-analysis examined the association of *Neisseria gonorrhoea* (responsible for gonorrhoea), *Treponema pallidum* (syphilis), *Chlamydia trachomatis* (chlamydia), *Trichomonas vaginalis* (trichomoniasis), *Ureaplasma urealyticum*, *Mycoplasma hominis*, Herpes Simplex Virus types 1 and 2, Human Herpes Virus 8, and Cytomegalovirus with the development and progression of PCa [19]. Men infected with gonorrhoea had a 20% increased risk of developing PCa. Moreover, while syphilis presented a significant association with PCa risk, none of the other STDs demonstrated relevance for the development and progression of the disease. Other authors suggested that only gonorrhoea and the human papillomavirus (HPV) were strongly linked to PCa risk [143]. In a population-based case-control study of black men, gonorrhoea and history of prostatitis increased the odds of developing PCa. In addition, men who have or have had more than one STD have an accentuated risk of PCa, which is consistent with studies in white men [18]. These findings suggest that STDs are a robust risk factor for PCa. However, the risk associated with each specific STD is different.

## 4. Impact of Inflammation on PCa

One of the potential risk factors for PCa that has gained relevance is the development of chronic inflammation in the prostate. Several factors, including viral or bacterial infections, dietary factors, hormonal changes, urine reflux, or physical injuries, can contribute to prostate inflammation [148,149]. Chronic inflammation has been identified as a major cause of approximately 20% of human cancers [150]. In the prostate microenvironment, it can promote alterations that facilitate cancer progression through proliferation, cell survival, evasion of immune surveillance, tissue remodelling, production of angiogenic factors, metastatic spread, and resistance to therapeutic agents [149]. In early prostate carcinogenesis, inflammation can be identified by proliferative inflammatory atrophy (PIA). This is a lesion with activated inflammatory immune cells that can invade the peripheral zone of the prostate, where most cancers occur [151]. These lesions can proliferate at a high rate, possibly in response to cellular damage caused by inflammation, upregulation of the apoptosis regulator B-cell lymphoma 2 (Bcl-2), and expression of the proto-oncogene MYC [152]. This has been identified as a precursor of PIN, which in turn is a precursor of BPH, and PCa [150].

Since inflammation has been demonstrated to be a potentially major factor in the development of PCa, the role of inflammatory markers has gained attention (Table 1). Chemokines and cytokines play a crucial role in PCa, by promoting tumour cell proliferation, the epithelial–mesenchymal transition, angiogenesis, and metastasis.

However, a specific chemokine may have pro-inflammatory or anti-inflammatory effects, depending on the circumstances. For instance, the cluster of differentiation 184 (CD184) is upregulated in PCa, but one or more of its ligands (stromal cell-derived factor 1 (SDF-1) and ubiquitin) have antiapoptotic effects [153]. In a case-control study, a group of circulating inflammatory markers were identified as possible contributors to PCa pathophysiology, including chemokine (C-X3-C motif) ligand 1 (CX3CL1), interleukin-10 (IL-10), platelet-derived growth factor-BB (PDGF-BB) (inverse associations), Chemokine (C-C motif) ligand 21 (CCL21), and Chemokine (C-C motif) ligand 11 (CCL11) (positive associations) [154].

As inflammation is influenced by numerous molecules, it is crucial to evaluate these and assess whether they can be novel biomarkers for a better understanding of PCa.

## 5. Identification of Novel Biomarkers, including Lifestyle-Associated Biomarkers

As PSA screening is the main diagnostic tool currently available for PCa and it has been associated with several limitations, including disease overdiagnosis and overtreatment, there is an urgent need for novel biomarkers. However, the challenges involved in the development of new ideal markers are vast. Biomarkers should be specific for the disease and should not be expressed in other tissues or tumours. Moreover, the collection method should be noninvasive, and biomarkers should be suitable for use in large-scale screening programmes. Additionally, an ideal biomarker should distinguish not only between patients, with and without PCa, but also between clinically significant tumours and other benign conditions, such as BPH [155]. For the noninvasive collection, body fluids, such as seminal fluid and urine, have been suggested [156].

Two potential biomarkers have been identified, the blood-based Prostate Health Index (PHI) and urinary prostate cancer antigen 3 (PCA3). PHI combines total PSA, free PSA (fPSA), and the PSA isoform p2PSA. Men at higher risk of significant PCa have higher levels of total PSA and p2PSA and lower levels of fPSA [157]. PHI is also associated with a biopsy Gleason score above 7 and it may have a role in monitoring men on active surveillance [157]. PCA3 is a prostate-specific noncoding mRNA that is detectable in urine and it was overexpressed in PCa tissue compared to normal prostate tissue [158,159]. Unlike PSA, PCA3 expression is less influenced by the patient’s age, prostate volume, inflammation, trauma, or prior biopsies [156,159]. Both PHI and PCA3 were able to predict PCa, but studies disagreed on accuracy [160,161,162]. Moreover, both also improved the prediction of tumour stage and tumour volume [163,164].

The 4K score is a test that combines a panel of four kallikrein proteins: total PSA, fPSA, intact PSA, and human kallikrein-related peptidase 2 (hK2) [165]. It uses an algorithm that takes into account the patient’s age, DRE, and history of previous biopsies [166]. Thus, it can provide a higher accuracy of the individual patient’s risk of high-grade PCa, compared to the one using PSA and fPSA [167]. As it is a more personalised test, it may prevent unnecessary biopsies and it can predict metastases up to 20 years ahead with higher precision [166,167].

A recent study investigated the possibility of exploring novel biomarkers through metabolic profiling of urine [168,169]. Indeed, metabolomics has been applied to different types of samples, including prostate tissue [170], cell lines [171], and serum [172], with the ultimate goal of finding novel diagnostic biomarkers. Regarding urine, guanidinoacetate, phenylacetylglycine, and glycine were appointed as potential candidate markers for PCa as their levels could be distinguished between PCa patients and healthy subjects. Another study examined exosomes in African American men compared with Caucasian men because PSA screening is not as effective in African American men [173,174]. Exosomes are small vesicles that are originated from endosomes and secreted into the extracellular milieu after the fusion of multivesicular endosomes with the plasma membrane. Their potential for diagnosis and prognosis has been suggested. Filamin A [175], Vitamin D-binding protein [176], and Afamin [177] have already been studied as biomarkers in breast cancer, pancreatic cancer, and ovarian cancer, respectively, and have also been identified in exosomes from African Americans with PCa [178].

Other potential biomarkers include miRNAs, such as miR-21, miR-182, and miR-101. miR-21 is highly expressed in solid tumours, including in prostate tumours [179,180,181,182]. This miRNA reduced the expression of Programmed cell death 4 (PDCD4), a suppressor of tumourigenesis and tumour progression, by expressing IL-6 in PCa cells [183]. The miR-183-96-182 cluster was reported to be overexpressed in PCa and miR-182 promotes PCa cell proliferation and invasion by targeting multiple genes [184,185,186,187]. Hypoxia is a hallmark of PCa with a poor prognosis and it enhances the presence of bone metastases [188,189,190]. Thus, the involvement of miR-182 in hypoxia adaptation and/or angiogenesis was evaluated. Hypoxia-activated miR-182 inhibited the negative regulators of the hypoxia-inducible 1α factor (HIF-1α) signalling pathway, hypoxia-inducible factor prolyl hydroxylase 2 (PHD2), and factor inhibiting HIF-1 (FIH-1), and increased HIF-1α signalling in PCa [191]. miR-182 could be a potential target for PCa as it encourages the irreversible activation of the HIF-1α pathway and the stable switching of the cellular state for tumour growth and angiogenesis under hypoxic conditions [191]. Finally, miRNA-101 is downregulated in PCa compared with normal tissues [192]. The ability to target COX-2 in several cancers has also been reported for miRNA-101 [193]. This miRNA was also able to inhibit COX-2 protein expression, decreasing the proliferation and growth of PCa cells in vitro and in vivo [193]. Thus, these three miRNAs may be potential biomarkers for PCa.

**Figure 1:**
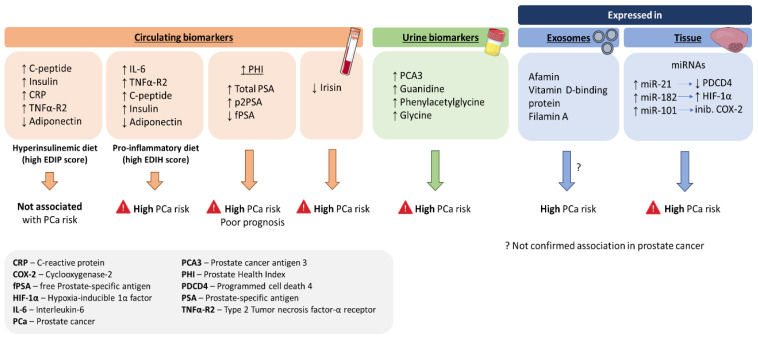
Possible novel biomarkers linked with PCa research and their association with PCa risk.

Several molecules, associated with lifestyle, have been used to evaluate PCa risk, and the examination of dietary patterns has also been considered an appealing approach (Figure 1). Hyperinsulinemia and inflammation are two interrelated biological pathways that have been linked with PCa risk [194,195]. Therefore, dietary patterns that directly influence these biological pathways may be more predictive of PCa risk. A study from 2020 [196] assessed circulating biomarkers such as C-reactive protein (CRP), adiponectin, interleukin-6 (IL6), type 2 TNF-α receptor (TNFα-R2), c-peptide, and insulin. The main goal was to evaluate these biomarkers using two different indexes: (i) the Empirical Dietary Index for Hyperinsulinemia (EDIH) score to assess the hyperinsulinemia potential of the diet [197] and (ii) the Empirical Dietary Inflammatory Pattern (EDIP) score to confirm the inflammatory potential of the diet [198]. Both indexes’ score predicted relevant biomarker concentrations to their respective patterns of dietary insulinemic and inflammatory potential. Moreover, the EDIH predicted future PCa risk, especially for high-grade PCa, suggesting a dietary pattern for PCa prevention.

Moreover, irisin, a myokine/adipokine, synthesised in many tissues, including skeletal muscle and fat cells, and that participates in the regulation of lipid and glucose metabolism, has also been studied to be a potential lifestyle-associated biomarker in PCa. As it regulates fat metabolism, it plays a crucial role in the arising and development of obesity, obesity-related insulin resistance, diabetes, non-alcoholic fatty liver disease, and other metabolic diseases [199,200]. In a recent prospective study, serum irisin levels were shown to be significantly lower in patients with PCa [201]. In recent years, many putative biomarkers for PCa have emerged that still need robust validation.

## 6. Conclusions

As PCa aetiology is still under investigation, it is necessary to establish the link between inflammation and general lifestyle in the progression of PCa. By identifying this association, it would be possible to adopt preventive measures, such as different diets or different activity behaviours. This is extremely important as it could help appraise the evolution of chronic inflammation and, consequently, PCa. Studying the impact of lifestyle and inflammation on PCa may open new avenues to identify a suitable biomarker for the diagnosis or treatment follow-up of PCa. Moreover, they could complement PSA screening, and enable the identification of men at higher risk of developing a severe form of the disease, which is imperative to prevent lethal PCa. Furthermore, novel biomarkers would prevent patients from being exposed to aggressive and unnecessary treatments.

## Figures and Tables

**Table 1:** Inflammation markers and their targeted processes in cancer (adapted from Archer M. et al. [149]).

Inflammation Markers	Targeted Processes
Interleukin-1 (IL-1)	Proliferation, survival, therapeutic resistance
Interleukin-6 (IL-6)	Proliferation, survival, anoikis resistance, metastasis, therapeutic resistance
Interleukin-8 (IL-8)	Proliferation, survival, angiogenesis, therapeutic resistance
Interleukin-23 (IL-23)	Therapeutic resistance
Nuclear factor kappa-light-chain-enhancer of activated B cells (NF-kB)	Anoikis resistance, metastasis, therapeutic resistance
C-C Motif Chemokine Ligand 2 (CCL-2)	Pro-tumour immunity, metastasis, therapeutic resistance
Transforming growth factor-beta (TGF-β)	Pro-tumour immunity, angiogenesis, epithelial-mesenchymal transition (EMT), metastasis, therapeutic resistance
Tumour necrosis factor-alpha (TNF-α)	Survival, EMT, anoikis resistance

## Data Availability

Not applicable.
